# Association of the triglyceride-glucose index with all-cause and cardiovascular mortality in patients with cardiometabolic syndrome: a national cohort study

**DOI:** 10.1186/s12933-024-02152-y

**Published:** 2024-02-24

**Authors:** Quanjun Liu, Yeshen Zhang, Shuhua Chen, Hong Xiang, Jie Ouyang, Huiqin Liu, Jing Zhang, Yanfei Chai, Zishun Zhan, Peng Gao, Xiao Zhang, Jianing Fan, Xinru Zheng, Zhihui Zhang, Hongwei Lu

**Affiliations:** 1 Health Management Center, The Third Xiangya Hospital of Central South University, Changsha, China; 2 Department of Cardiology, The Third Xiangya Hospital of Central South University, No. 138, Tongzipo Road, Yuelu District, Changsha, China; 3 Department of Biochemistry, School of Life Sciences of Central, South University, Changsha, China; 4 Center for Experimental Medicine, The Third Xiangya Hospital of Central South University, Changsha, China

**Keywords:** Triglyceride-glucose index, Mortality, Insulin resistance, Cardiometabolic syndrome, NHANES

## Abstract

**Objective:**

This study aimed to evaluate the association of triglyceride-glucose (TyG) index with all-cause and cardiovascular mortality risk among patients with cardiometabolic syndrome (CMS).

**Methods:**

We performed a cohort study of 5754 individuals with CMS from the 2001–2018 National Health and Nutrition Examination Survey. The TyG index was calculated as Ln [fasting triglycerides (mg/dL) × fasting glucose (mg/dL)/2]. Multivariate Cox proportional hazards regression models assessed the associations between TyG index and mortality . Non-linear correlations and threshold effects were explored using restricted cubic splines and a two-piecewise Cox proportional hazards model.

**Results:**

Over a median follow-up of 107 months, 1201 all-cause deaths occurred, including 398 cardiovascular disease-related deaths. The multivariate Cox proportional hazards regression model showed a positive association between the TyG index and all-cause and cardiovascular mortality. Each one-unit increase in the TyG index was associated with a 16% risk increase in all-cause mortality (HR: 1.16, 95% CI 1.03, 1.31, *P* = 0.017) and a 39% risk increase in cardiovascular mortality (HR: 1.39, 95% CI 1.14, 1.71, *P* = 0.001) after adjusting for confounders. The restricted cubic splines revealed a U-shaped association between the TyG index and all-cause (*P* for nonlinear < 0.001) and cardiovascular mortality (*P* for nonlinear = 0.044), identifying threshold values (all-cause mortality: 9.104; cardiovascular mortality: 8.758). A TyG index below these thresholds displayed a negative association with all-cause mortality (HR: 0.58, 95% CI 0.38, 0.90, *P* = 0.015) but not with cardiovascular mortality (HR: 0.39, 95% CI 0.12, 1.27, *P* = 0.119). Conversely, a TyG index exceeding these thresholds was positively associated with all-cause and cardiovascular mortality (HR: 1.35, 95% CI 1.17, 1.55, *P* < 0.001; HR: 1.54, 95% CI 1.25, 1.90, *P* < 0.001, respectively). Notably, a higher TyG index (≥ threshold values) was significantly associated with increased mortality only among individuals aged under 55 compared to those with a lower TyG index (< threshold values).

**Conclusions:**

The TyG index demonstrated a U-shaped correlation with all-cause and cardiovascular mortality in individuals with CMS. The thresholds of 9.104 and 8.758 for all-cause and cardiovascular mortality, respectively, may be used as intervention targets to reduce the risk of premature death and cardiovascular disease.

## Background

The prevalence of cardiometabolic syndrome (CMS) shows a tendency to increase, mirroring the increases observed in obesity and type 2 diabetes, attributed to the prevalence of high-calorie, low-fiber diets, decreased physical activity, and prolonged sedentary behavior [1–3]. According to the National Health and Nutrition Examination Survey (NHANES) data spanning from 1988–1994 to 2007–2012, the prevalence of CMS among adults in the United States (US) surged from 25.3% to 34.2% [4]. Various prospective studies have highlighted that CMS not only heightens the risk of cardiovascular disease (CVD) and diabetes but also significantly amplifies cardiovascular mortality and all-cause mortality [5–9]. Consequently, CMS presents a substantial global challenge in public health and clinical realms. Thus, an urgent need exists to evaluate the population at high risk of death among CMS patients and formulate clinical strategies to avert adverse events.

Individuals with higher insulin resistance (IR) are prone to various metabolic disorders, such as high blood sugar, abnormal lipid levels, and hypertension. IR has been confirmed as a predictive factor for cardiovascular diseases and adverse cardiovascular events [5]. Additionally, IR serves as the primary pathological mechanism of CMS [11, 12], prevailing in most CMS patients and strongly correlating with CVD risk [13]. To date, there remains a notable absence of clinically feasible and accurate methods for assessing IR. The gold standard for assessing IR, including the hyperinsulinemic-euglycemic clamp and intravenous glucose tolerance test, is characterized by their prohibitively high costs and invasiveness, rendering them less applicable in extensive epidemiological surveys [14]. Presently, the widely employed index for evaluating IR is the Homeostasis Model Assessment of Insulin Resistance (HOMA-IR) [15]; however, fasting insulin measurements have not gained widespread usage in clinical settings.

The triglyceride-glucose (TyG) index, a composite marker combined with fasting triglycerides and glucose for assessing IR, effectively substitutes conventional IR markers in diagnosing CMS [16]. The TyG index offers easier accessibility and cost-effectiveness compared to traditional IR indicators. Furthermore, this index has been validated to correlate with adverse cardiovascular and metabolic-related events [17–20]. However, there has not been research delving into the relationship between the TyG index and all-cause mortality, as well as cardiovascular mortality among patients with CMS.

Our study aims to evaluate whether the TyG index correlates with the risk of all-cause mortality and cardiovascular mortality among individuals with CMS, utilizing data from the NHANES cohort spanning 2001–2018.

## Materials and methods

### Study population and design

NHANES is a cross-sectional, multistage, stratified, clustered probability survey conducted by the National Center for Health Statistics (NCHS) at the Centers for Disease Control and Prevention [21]. The survey protocol received approval from the NCHS institutional review board, and all respondents provided written informed consent. Accessible NHANES data for this analysis can be found at https://www.cdc.gov/nchs/nhanes.

**Fig. 1:**
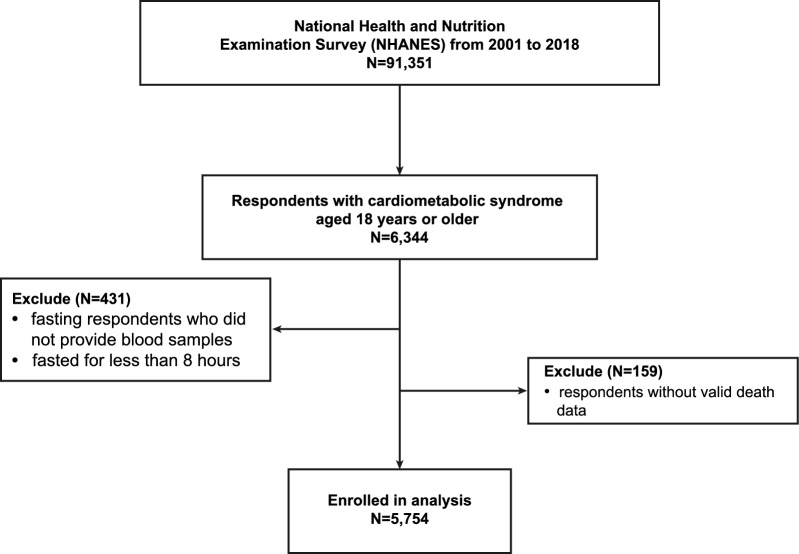
Screening flow of respondents

This is a national cohort study of NHANES respondents with CMS from 2001 to 2018, assessed in accordance with the criteria of the National Cholesterol Education Program (NCEP) Adult Treatment Panel III (ATP III) [22]. CMS was defined as meeting three or more of the following criteria: (1) waist circumference of ≥ 102 cm in men and ≥ 88 cm in women; (2) high circulating triglycerides (TG) ≥ 150 mg/dL; (3) low high-density lipoprotein cholesterol (HDL-C) < 40 mg/dL for men and < 50 mg/dL for women; (4) high fasting blood glucose ≥ 110 mg/dL; (5) diagnosis of arterial hypertension (≥ 130/ ≥ 85 mmHg). After excluding respondents who did not provide blood samples or fasted for less than 8 h (n = 431) and those without valid death data (n = 159), 5,754 individuals from the NHANES dataset with CMS were included in this analysis (Fig. 1).

### Measurement of the TyG index

The TyG index, calculated as Ln [fasting triglycerides (mg/dL) × fasting glucose (mg/dL)/2], utilized triglycerides and glucose levels from sample persons fasting for at least 8 h but less than 24 h [23]. Fasting blood triglycerides were measured using three different analyzers (Roche Hitachi 717/912, Roche modular P chemistry, and Roche/Hitachi Cobas 6000). Fasting blood glucose (FBG) measurement utilized two instruments (Roche C501 from 2001 to 2015 and Roche C311 from 2015 to 2018). Considering different instruments for these indicators was not necessary under NHANES analysis guidelines. Respondents were categorized into four groups (Q1, Q2, Q3, Q4) based on the TyG index quartiles.

### Demographic characteristics and other covariate

Race/ethnicity (non-Hispanic white, non-Hispanic black, Hispanic, other race) were categorized based on the survey design. Education level was simplified into below high school (less than 11th grade), high school graduate or general educational development test (GED) (high school Grad/GED), and some college or above (AA degree or College or above). In addition, marital status was divided into married or living with a partner, never married, widowed/divorced/separated, and never married. The Poverty-Income Ratio (PIR) served as an index of income related to federally established poverty thresholds, accounting for economic inflation and family size. Nicotine exposure, alcohol use, physical activity, history of diabetes, history of CVD, history of hypertension, and history of cancer were obtained via self-report questionnaires. The nicotine exposure has been classified as never smoker, former smoker, or current smoker. The alcohol use was classified into four categories: non-drinker, 1–5 drinks per month, 5–10 drinks per month, and more than 10 drinks per month. Moderate and vigorous physical activity duration was reported by respondents during leisure time. Respondents with physical inactivity if they engaged in moderate-intensity physical activity for < 150 min per week, vigorous-intensity physical activity for < 75 min per week, or an equivalent combination of the two [24]. History of CVD included self-reported angina pectoris, congestive heart failure, coronary heart disease, heart attack, and stroke.

Blood pressure, weight, height, and waist circumference measurements were acquired using standard methods in the mobile examination center. Body mass index (BMI) was calculated as weight/height^2^ from these measurements. Clinical indicators such as TG, total cholesterol (TC), low-density lipoprotein cholesterol (LDL-C), HDL-C, FBG, Hemoglobin A1c (HbA1c), Fasting blood insulin (FBI), albumin (ALB), alanine aminotransferase (ALT), aspartate aminotransferase (AST), blood urea nitrogen (BUN), glutamyl transpeptidase (GGT), lactic dehydrogenase (LDH), total bilirubin (TBIL), serum uric acid (SUA), and serum creatinine concentration (SCR) were measured in the NHANES laboratory. eGFR was calculated using the chronic kidney disease-epidemiology collaboration (CKD-EPI) equation, and chronic kidney disease (CKD) was defined as an eGFR of 15–59 mL/min/1.73 m^2^ [21].

### Ascertainment of mortality

The study encompassed all-cause and cardiovascular mortality as endpoints [25]. All-cause mortality was defined as death from heart diseases, malignant neoplasms, and all other causes. Cardiovascular mortality was defined as the death attributed to heart diseases (ICD-10 codes I00–I09, I11, I13, I20–I51) and cerebrovascular diseases (ICD-10 codes I60–I69), according to the International Classification of Diseases (10th Clinical Modification (ICD-10) system) [26]. Mortality data from NHANES were linked to death certificate data from the National Death Index of the NCHS until December 31, 2019, employing a probability matching algorithm. The helpful website for additional information regarding mortality variables is available at: (https://www.cdc.gov/nchs/data-linkage/mortality.htm).

### Statistical analysis

The NHANES is a multistage, stratified, probability-based survey that oversamples certain groups [27]. To account for unequal sampling probability and nonresponses, data for all respondents has been weighted using the recommended NHANES exam weights and fasting subsample weights. Participants were divided into TyG index quartiles (Q1–Q4) for analyses. Mean ± standard deviation (SD) was used to present continuous variables, which were compared using the Wilcoxon rank-sum test based on the study design. Frequency (percentages) was used to present categorical variables, which were compared using Chi-square tests.

Multivariate Cox proportional hazards regression models were utilized to assess the associations between the TyG index and mortality, adjusting for potential confounders. Due to the large number of risk factors investigated in this study, only relevant demographic characteristics and traditional factors associated with the TyG index and deaths were included in the multivariate Cox regression analysis. Hazard ratios (HRs) were calculated across three models: an unadjusted model (Model 1), an age, gender, and race/ethnicity-adjusted model (Model 2), and a comprehensive adjustment for potential confounders (Model 3), encompassing age, gender, race, poverty ratio, marital status, education levels, BMI, nicotine exposure, alcohol use, and physical inactivity.

To explore potential nonlinear relationships between the TyG index and mortality, restricted cubic spline regression for HR was employed. Upon confirmation of a nonlinear relationship, we estimate the threshold value using the maximum likelihood method. A two-piecewise Cox proportional risk model was applied on both sides of the inflection point to further investigate the relationship between the TyG index and the risk of all-cause and cardiovascular mortality.

Missing covariates were addressed through a multilevel imputation approach designed for survey data [28]. The results obtained from this imputation were consistent with analyses that excluded participants with missing covariates. Subsequently, stratification and interaction analyses were performed by gender, age, race, medicine history, physical activity, nicotine exposure, and alcohol use. All analyses were executed using R software (version 4.3.1) and EmpowerStats software (www.empowerstats.com, X&Y solutions, Inc. Boston MA, USA) with a significance level set at a two-tailed alpha of 0.05.

## Results

### Baseline characteristics of study participants

Based on the quartile of the TyG index within the study, the baseline characteristics of 5,754 respondents are shown in Table 1. Compared with those in the lowest quartile, respondents with a higher TyG index are more likely to be men, non-Hispanic whites, current smokers, and have hypertension, diabetes, and CVD. They generally have a lower BMI, BP, HDL, and LDH but higher HbA1c, FBG, FBI, TG, TC, LDL, ALB, ALT, AST, BUN, GGT, TBIL, and SUA (all *P* < 0.05). Moreover, respondents with an elevated TyG index have a heightened risk of both all-cause and cardiovascular mortality in contrast to those with a lower TyG index (all-cause mortality: 20.06% vs 15.16%, P = 0.014; cardiovascular mortality: 7.14% vs 4.06%, *P* = 0.011).

**Table 1:** Baseline characteristics according to the TyG index quartiles

Characteristics	Quantile of the TyG index					P value
	Overall	Q1 (7.36, 8.94)	Q2 (8.95, 9.21)	Q3 (9.22, 9.57)	Q4 (9.58, 13.40)	
N (%)	5754	1485	1388	1410	1471	
Age, years, mean (SD)	52.83 (15.88)	53.06 (17.01)	52.36 (15.99)	53.33 (15.97)	52.57 (14.42)	0.429
Gender, n (%)						< 0.001
Male	2743 (49.32)	641 (44.56)	575 (42.93)	680 (48.40)	847 (61.41)	
Female	3011 (50.68)	844 (55.44)	813 (57.07)	730 (51.60)	624 (38.59)	
BMI, kg/m^2^, mean (SD)	33.01 (6.70)	33.71 (7.40)	32.83 (6.51)	32.94 (6.58)	32.57 (6.18)	0.042
Waist circumference, cm, mean (SD)	110.66 (14.60)	111.60 (15.16)	109.59 (14.04)	110.74 (15.04)	110.70 (14.07)	0.075
Race, n (%)						< 0.001
Non-Hispanic White	2681 (70.97)	594 (65.00)	685 (73.23)	722 (74.01)	680 (71.65)	
Non-Hispanic Black	903 (8.84)	421 (16.52)	190 (7.33)	144 (5.39)	148 (6.09)	
Hispanic	1265 (9.19)	246 (7.69)	296 (8.95)	326 (9.29)	397 (10.84)	
Multiracial/other	905 (11.00)	224 (10.78)	217 (10.49)	218 (11.31)	246 (11.42)	
PIR, mean (SD)	2.82 (1.59)	2.82 (1.63)	2.84 (1.56)	2.83 (1.58)	2.79 (1.60)	0.934
Education level, n (%)						0.159
Below high school	1,929 (21.74)	445 (18.72)	464 (21.45)	493 (23.72)	527 (23.09)	
High school graduate or GED	1,396 (27.28)	372 (28.02)	318 (25.99)	368(28.59)	338 (26.53)	
Some college or above	2,429 (50.97)	668 (53.26)	606 (52.56)	549 (47.69)	606 (50.38)	
Marital status, n (%)						0.739
Married or living with a partner	3601 (66.23)	888 (65.05)	881 (66.94)	889 (66.85)	943 (66.10)	
Never married	641 (11.38)	200 (12.93)	138 (10.49)	152 (10.67)	151 (11.41)	
Widowed, divorced, or separated	1,512 (22.39)	397 (22.02)	369 (22.57)	369 (22.48)	377 (22.49)	
Nicotine exposure, n (%)						0.004
Never	2811 (48.66)	784 (52.53)	725 (51.26)	631 (46.27)	671 (44.56)	
Former	1749 (30.05)	424 (29.11)	390 (26.58)	467 (32.95)	468 (31.57)	
Now	1194 (21.29)	277 (18.36)	273 (22.16)	312 (20.79)	332 (23.87)	
Alcohol use, n (%)						0.190
Non-drinker	2108 (32.69)	581 (34.49)	534 (33.59)	498 (33.56)	495 (29.13)	
1–5 drinks/month	2713 (47.83)	682 (47.82)	640 (46.77)	678 (48.23)	713 (48.51)	
5–10 drinks/month	312 (6.62)	64 (4.83)	74 (7.14)	81 (6.29)	93 (8.23)	
> 10 drinks/month	621 (12.85)	158 (12.85)	140 (12.50)	153 (11.92)	170 (14.12)	
Physical activity time, min/week, mean (SD)	220.85 (427.59)	206.53 (337.09)	220.01 (348.96)	224.26 (470.88)	232.65 (523.97)	0.285
Physical inactivity, n (%)	3,865 (65.11)	998 (65.80)	915 (62.93)	943 (64.43)	1,009 (67.28)	0.334
Medical history, n (%)						
Hypertension	3105 (51.35)	865 (53.53)	707 (47.34)	741 (51.75)	792 (52.78)	0.046
Diabetes	1463 (21.57)	306 (14.65)	235 (14.58)	334 (21.42)	588 (35.66)	< 0.001
CVD	964 (14.50)	269 (15.01)	193 (11.30)	243 (15.39)	259 (16.30)	0.021
CKD	704 (9.78)	187 (10.38)	161 (8.82)	190 (10.87)	166 (9.04)	0.325
Cancer	659 (11.79)	157 (11.45)	167 (11.73)	186 (12.63)	149 (11.37)	0.833
SBP, mmHg, mean (SD)	131.47 (18.35)	135.29 (18.19)	129.78 (18.70)	130.62 (18.52)	130.17 (17.44)	< 0.001
DBP, mmHg, mean (SD)	73.86 (13.84)	75.04 (14.61)	73.05 (12.79)	73.57 (13.58)	73.78 (14.23)	0.005
Laboratory measurements, mean (SD)						
HbA1c, %	6.09 (1.34)	5.78 (0.78)	5.75 (0.78)	5.93 (0.98)	6.89 (2.03)	< 0.001
FBG, mmol/L	6.85 (2.51)	6.17 (1.09)	6.11 (1.17)	6.50 (1.53)	8.64 (4.01)	< 0.001
FBI, pmol/L	118.95 (125.80)	111.17 (115.78)	107.14 (111.39)	118.90 (97.99)	138.64 (165.34)	< 0.001
TG, mmol/L	2.42 (2.08)	1.18 (0.39)	1.85 (0.31)	2.37 (0.47)	4.27 (3.41)	< 0.001
TC, mmol/L	5.24 (1.21)	4.72 (1.01)	5.19 (1.07)	5.32 (1.09)	5.72 (1.40)	< 0.001
LDL, mmol/L	3.08 (0.98)	2.96 (0.89)	3.20 (0.99)	3.13 (1.00)	3.02 (1.04)	< 0.001
HDL, mmol/L	1.12 (0.32)	1.22 (0.40)	1.14 (0.29)	1.11 (0.26)	0.99 (0.25)	< 0.001
ALB, g/L	41.82 (3.28)	41.09 (3.17)	41.94 (3.33)	42.08 (3.15)	42.17 (3.36)	< 0.001
ALT, IU/L	30.14 (37.61)	27.41 (17.94)	28.06 (18.04)	32.37 (67.73)	32.74 (20.27)	< 0.001
AST, IU/L	26.35 (14.99)	25.38 (13.24)	25.50 (16.57)	26.69 (15.10)	27.84 (14.76)	< 0.001
BUN, mmol/L	5.10 (2.18)	5.08 (2.29)	4.92 (2.04)	5.11 (2.14)	5.31 (2.25)	< 0.001
GGT, IU/L	37.89 (55.90)	31.46 (37.55)	33.19 (37.80)	35.59 (35.56)	51.34 (90.32)	< 0.001
LDH, IU/L	137.44 (30.81)	141.89 (33.16)	138.17 (30.16)	134.57 (27.01)	135.10 (32.02)	< 0.001
TBIL, umol/L	11.40 (4.82)	10.94 (4.87)	11.27 (4.61)	11.87 (4.97)	11.51 (4.77)	< 0.001
SUA, IU/L	355.80 (87.28)	348.42 (87.07)	350.94 (83.76)	360.75 (84.64)	363.12 (92.58)	< 0.001
SCR, umol/L	78.58 (33.10)	77.87 (28.41)	76.96 (27.43)	78.49 (27.16)	81.03 (45.60)	0.076
eGFR, mL/min/1.73 m^2^	90.15 (22.60)	90.92 (23.64)	90.07 (21.70)	89.17 (23.00)	90.42 (21.99)	0.288
TyG index, mean (SD)	9.28 (0.61)	8.59 (0.31)	9.08 (0.07)	9.37 (0.10)	10.06 (0.50)	< 0.001
All-cause mortality, n (%)	1,201 (17.15)	279 (15.16)	275 (15.48)	302 (17.91)	345 (20.06)	0.014
Cardiovascular mortality, n (%)	398 (5.42)	86 (4.06)	93 (4.76)	96 (5.70)	123 (7.14)	0.011

Data presented as mean (standard deviation, SD) for continuous and no. (%) values for categorical

Chi-squared test with Rao and Scott’s second-order correction; Wilcoxon rank-sum test for complex survey samples

*TyG index* triglyceride-glucose index, *PIR* poverty-income ratio, *GED* general educational development test, *CKD* chronic kidney disease, *CVD* cardiovascular disease, *SBP* systolic blood pressure, *DBP* diastolic blood pressure, *HbA1c* hemoglobin A1c, *FBG* fasting blood glucose, *FBI* fasting blood insulin, *TG* triglyceride, *TC* total cholesterol, *LDL* low-density lipoprotein, *HDL* high-density lipoprotein, *ALB* albumin, *ALT* alanine aminotransferase, *AST* aspartate aminotransferase, *BUN* blood urea nitrogen, *GGT* glutamyl transpeptidase, *LDH* lactic dehydrogenase, *TBIL* total bilirubin, *SUA* serum uric acid, *SCR* serum creatinine, *eGFR* estimated glomerular filtration rate

### Association of TyG index with mortality

Cox proportional hazard analysis was conducted to assess the association between TyG index levels and mortality risk in respondents with CMS. Over a median follow-up of 107 months, a total of 1201 all-cause deaths and 398 cardiovascular deaths were recorded. Table 2 presents results from three Cox regression model analyses. Models 1 and 2 indicate upward trends between the TyG index and both all-cause and cardiovascular mortality (*P* for trend < 0.05). After adjusting for age, gender, race, education level, family income-poverty ratio, marital status, physical inactivity, BMI, nicotine exposure, and alcohol use in Model 3, the HRs and 95% confidence intervals (CIs) were 1.00 (reference), 0.92 (0.75, 1.12), 0.95 (0.78, 1.15), and 1.19 (0.98, 1.45) for the Q1, Q2, Q3, and Q4 groups, respectively, for all-cause mortality (*P* for trend = 0.077). Correspondingly, the HRs and 95% CIs were 1.11 (0.79, 1.56), 1.19 (0.82, 1.73), and 1.68 (1.22, 2.31) for the Q2, Q3, and Q4 groups, respectively, in relation to cardiovascular mortality (*P* for trend = 0.003) compared with the Q1 group. The continuous models indicate that every one-unit increase in the TyG index was associated with an increased risk of 1.16 (1.03, 1.31) for all-cause mortality and 1.39 (1.14, 1.71) for cardiovascular mortality after adjusting for confounding factors (Table 3).

**Table 2:** Associations between the TyG index and all-cause and cardiovascular mortality in patients with cardiometabolic syndrome

	Quartiles of the TyG index				*P* for trend
	Q1	Q2	Q3	Q4	
All-cause mortality					
Number of deaths	279	275	302	345	–
Model 1	Ref.	0.85 (0.69, 1.04)	1.03 (0.84, 1.27)	1.22 (0.99, 1.50)	0.027
Model 2	Ref.	0.96 (0.79, 1.16)	1.00 (0.82, 1.21)	1.29 (1.07, 1.56)	0.010
Model 3	Ref.	0.92 (0.75, 1.12)	0.95 (0.78, 1.15)	1.19 (0.98, 1.45)	0.077
Cardiovascular mortality					
Number of deaths	86	93	96	123	–
Model 1	Ref.	0.97 (0.67, 1.41)	1.23 (0.83, 1.83)	1.63 (1.14, 2.33)	0.005
Model 2	Ref.	1.14 (0.82, 1.60)	1.22 (0.84, 1.76)	1.79 (1.28, 2.51)	0.002
Model 3	Ref.	1.11 (0.79, 1.56)	1.19 (0.82, 1.73)	1.68 (1.22, 2.31)	0.003

Cox proportional hazard models were used to estimate HR and 95% CI

Model 1 was unadjusted, Model 2 was adjusted for age, race, and gender, and Model 3 was adjusted for age, gender, race, education level, family income-poverty ratio, marital status, physical inactivity, body mass index, nicotine exposure, and alcohol use

*HR* hazard ratio, *CI* confidence interval, *TyG index* triglyceride-glucose index, *CV* cardiovascular

**Table 3:** Threshold effect analysis of TyG index on all-cause and cardiovascular mortality in patients with cardiometabolic syndrome^a^

	HR (95% CI)	*P* value
All-cause mortality		
Fitting by the standard Cox proportional risk model	1.16 (1.03, 1.31)	0.017
Fitting by the two-piecewise Cox proportional risk model		
Inflection point	9.104	
TyG index < 9.104	0.58 (0.38, 0.90)	0.015
TyG index ≥ 9.104	1.35 (1.17, 1.55)	< 0.001
P for Log-likelihood ratio	< 0.001	
Cardiovascular mortality		
Fitting by the standard Cox proportional risk model	1.39 (1.14, 1.71)	0.001
Fitting by the two-piecewise Cox proportional risk model		
Inflection point	8.758	
TyG index < 8.758	0.39 (0.12, 1.27)	0.119
TyG index ≥ 8.758	1.54 (1.25, 1.90)	< 0.001
P for Log-likelihood ratio	0.001	

*HR* hazard ratio, *CI* confidence interval, *TyG index* triglyceride-glucose index

Cox proportional hazard models were used to estimate HR and 95% CI

^a^Adjusted for age, gender, race, education level, family income-poverty ratio, marital status, physical inactivity, BMI, nicotine exposure, and alcohol use

**Fig. 2:**
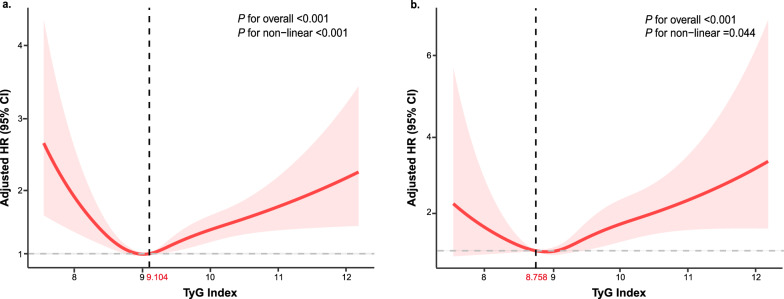
Multivariable adjusted spline curves for associations of the TyG index with all-cause (**a**) and cardiovascular mortality (**b**) in respondents with cardiometabolic syndrome. Hazard ratios adjusted for age (as a continuous variable), gender and race, poverty ratio (as a continuous variable), education levels, marital status, body mass index (as a continuous variable), nicotine exposure, alcohol use, and physical inactivity. The solid line and red area represent the estimated values and their corresponding 95% CI. *HR* hazard ratio, *CI* confidence interval, *TyG index* triglyceride-glucose index

Utilizing Cox proportional hazards regression models with restricted cubic splines, the association between the TyG index and all-cause and cardiovascular mortality was further examined. A non-linear dose–response relationship was observed between the TyG index and mortality incidence (Fig. 2). Intriguingly, adjusted smoothed plots depicted U-shaped associations between the TyG index and both all-cause (*P* for non-linear < 0.001, Fig. 2a) as well as cardiovascular mortality (*P* for non-linear = 0.044, Fig. 2b). Additionally, inflection points for all-cause and cardiovascular mortality were identified as 9.104 and 8.758, respectively (both *P* values for log-likelihood ratio < 0.001) (Table 3). Following adjustments for various factors, the risk of all-cause mortality decreased by 42% (HR: 0.58, 95% CI 0.38, 0.90) for each unit increase in TyG index below the threshold value and by 35% (HR: 1.35, 95% CI 1.17, 1.55) for each unit increase in TyG index above the threshold value (*P* = 0.015, *P* < 0.001, respectively). However, the TyG index below the threshold value did not significantly associate with the risk of cardiovascular mortality but exhibited a 54% (HR: 1.54, 95% CI 1.25, 1.90) increase in risk per unit increase above the threshold value (*P* = 0.119, *P* < 0.001, respectively).

### Stratified analyses

**Fig. 3:**
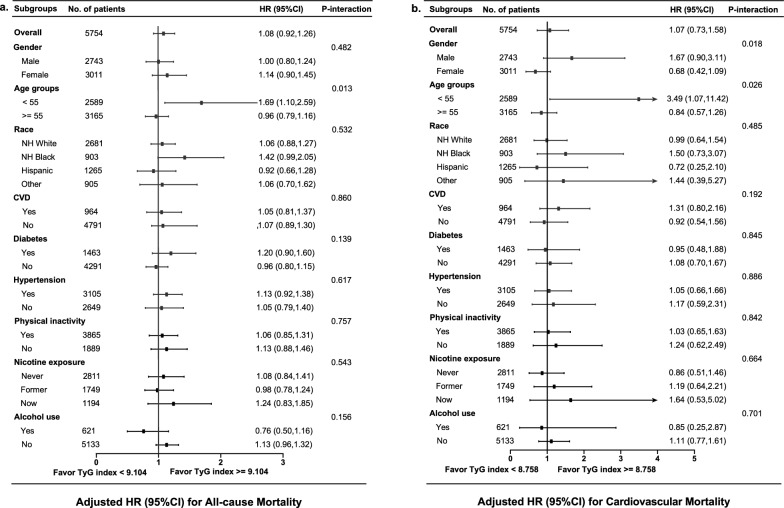
Stratified analyses of the associations between the TyG index and all-cause (**a**) and cardiovascular mortality (**b**) among respondents with cardiometabolic syndrome. Hazard ratios were estimated using a two-piecewise Cox proportional risk model on both sides of the inflection point (all-cause mortality: 9.104; cardiovascular mortality: 8.758) and adjusted for confounders. Alcohol use was defined as more than 10 drinks per month. *HR* hazard ratio, *CI* confidence interval, *TyG index* triglyceride-glucose index, *NH* non-Hispanic

To elucidate the survival advantage of a higher TyG index (all-cause mortality: ≥ 9.104; cardiovascular mortality: ≥ 8.758) compared to a lower TyG index (all-cause mortality: < 9.104; cardiovascular mortality: < 8.758) among respondents with CMS, stratification and interaction analyses were conducted for gender, age, race, medicine history, physical inactivity, nicotine exposure, and alcohol use (Fig. 3). Except for the age subgroup (all-cause mortality: *P*-interaction = 0.013; cardiovascular mortality: *P*-interaction < 0.026) and gender subgroup (cardiovascular mortality: *P*-interaction = 0.018), most subgroups did not exhibit significant interaction (*P*-interaction > 0.05). A higher TyG index correlated closely with increased all-cause and cardiovascular mortality in patients aged < 55 (all-cause mortality, HR: 1.69, 95% CI 1.10, 2.59, *P* < 0.05; cardiovascular mortality, HR: 3.49, 95% CI 1.07, 11.42, *P* < 0.05), while this association was not observed in patients aged ≥ 55 (all-cause mortality, HR: 0.96, 95% CI 0.79, 1.16, *P* > 0.05; cardiovascular mortality, HR: 0.84, 95% CI 0.57, 1.26, *P* > 0.05). Furthermore, gender influenced the relationship between the TyG index and cardiovascular mortality but was not found to be statistically significant in the subgroups.

## Discussion

To our knowledge, our study represents the first exploration of the association between the TyG index and all-cause mortality and cardiovascular mortality among individuals with CMS. Our research identified a U-shaped correlation between the TyG index and all-cause mortality and cardiovascular mortality, elucidating the threshold points (all-cause mortality: 9.104; CV mortality: 8.758). Specifically, a higher TyG index (≥ threshold values) was significantly associated with increased mortality among individuals aged < 55 compared to those with a lower TyG index. This study highlighted the TyG index as significant and may be helpful in identifying patients with CMS at high risk of mortality and guiding further detections and more aggressive treatments.

Prior studies have already established the significant predictive role of the TyG index for adverse events among healthy individuals and those with CVD [19, 20, 29]. The findings in our study demonstrated that the TyG index was positively associated with higher all-cause and cardiovascular mortality in patients with CMS, which indicated the usefulness of the TyG index in screening individuals who have increased mortality risk in such a population. CMS is acknowledged as an independent risk factor for CVD and diabetes [10], and elevated TyG index levels exhibit a distinct association with an increased likelihood of developing cardiovascular and diabetic conditions [30, 31]. Elevated TyG index levels might escalate the incidence of cardiovascular diseases and diabetes within the CMS population, consequently heightening overall mortality and cardiovascular mortality. Numerous prospective studies utilizing the HOMA-IR index to assess IR demonstrate that IR substantially increases individual diabetes or CVD risk in patients with CMS, suggesting that CMS does not equate to an insulin-resistant phenotype [32–34]. The TyG index, an easily accessible surrogate marker of IR [16, 35], is implicated in endothelial dysfunction, impaired cardiac autonomic function, chronic inflammation, and heightened sympathetic nervous system activity, thus accelerating the progression of cardiovascular diseases [36–40]. Recent studies underscore the critical role of IR, chronic inflammation, and neurohormonal activation as pivotal elements in the progression of CMS pathophysiology [10]. Consistent with previous studies, our study also found that the TyG index was positively correlated with traditional cardiovascular risk factors such as HbA1c, FBG, FBI, TG, and TC and negatively correlated with HDL-C [41]. These data support IR as an independent risk factor among CMS patients and suggest increased TyG index measurement could identify individuals at a heightened risk. Compared to the HOMA-IR index, the TyG index, incorporating fasting blood glucose and lipid parameters, is more easily obtainable, with fewer laboratory procedures and lower costs, making it more convenient for clinical application.

Consistent with prior research [20], our study confirmed the association of the TyG index with all-cause mortality and cardiovascular mortality, revealing a U-shaped relationship among patients with CMS. Specifically, a unit increase below the threshold is related to a 42% reduction in all-cause mortality. A cohort analysis concerning statin therapy has suggested elevated triglyceride levels were linked to an increased risk of cardiovascular disease events but a decreased risk of mortality [42]. Similarly, another study affirmed a J-shaped relationship between blood glucose levels and all-cause mortality or cardiovascular events, associating lower fasting blood glucose levels with increased adverse events [43]. Extremely low triglyceride and fasting glucose levels might indicate poor nutritional status. Additionally, hypoglycemia might trigger cardiac arrhythmias, thrombus formation, vascular inflammation, and vasoconstriction, leading to increased cardiovascular events or mortality [44, 45]. Therefore, maintaining an optimal TyG index level is crucial, as excessively high and low levels can lead to detrimental health outcomes. Notably, our study stratified CMS patients according to age, revealing that higher TyG index (all-cause mortality: ≥ 9.104; CV mortality: ≥ 8.758) exhibited a significant association with increased mortality compared to patients with lower TyG index (all-cause mortality: < 9.104; CV mortality: < 8.758), only in individuals aged under 55. This information provides the theoretical foothold for the application of the TyG index in the non-older population. This data might support using the TyG index in the non-older CMS population, emphasising the significance of managing the TyG index at lower levels for their health benefits.

The strengths of the study include its substantial sample size, long follow-up time, and the assessment of the dose–response relationship between the TyG index and mortality, identifying the inflection point in the U-shaped relationship in CMS patients. Nevertheless, we need to consider several potential limitations in our study. Firstly, our analysis involved participants from a single nation, potentially limiting the global applicability of our conclusions. Secondly, Due to the post hoc nature of the study, residual confounding elements may persist despite our efforts to control them. Thirdly, our study did not involve dynamic monitoring of the TyG index, precluding the determination of its long-term status. Additionally, our main exploration centered around the relationship between the TyG index and mortality, without comparing with other non-insulin-based IR indicators. Fourthly, due to the absence of specific information, we did not employ the latest definition to identify CMS patients, hindering the ability to ascertain the robustness of the study results through sensitivity analysis. However, the CMS definition utilized in this study has been thoroughly validated in previous research. Despite these limitations, our findings could extend our understanding of the association between the TyG index and mortality risk and provide new insights and clues for future studies into predicting adverse events among CMS patients.

## Conclusions

This cohort study demonstrated the relationship between the TyG index and both all-cause and cardiovascular mortality in individuals diagnosed with CMS. Notably, a U-shaped correlation was observed between the TyG index and all-cause as well as cardiovascular mortality. Adding the TyG index assessment will facilitate a more convenient and effective screening of individuals at high risk in CMS patients. Furthermore, the threshold can serve as an intervention target to mitigate the risk of premature mortality and cardiovascular diseases.

## Data Availability

The datasets that were used and evaluated in this study can be obtained from the corresponding author upon making a reasonable request.

## Abbreviations

ALB: Albumin
ALT: Alanine aminotransferase
AST III: Aspartate aminotransferase III
ATP: Adult treatment panel
BMI: Body mass index
BUN: Blood urea nitrogen
CDC: The Centers for Disease Control and Prevention
CI: Confidence interval
CKD: Chronic kidney disease
CKD-EPI: Chronic kidney disease-epidemiology collaboration
CV: Cardiovascular
CVD: Cardiovascular disease
DBP: Diastolic blood pressure
eGFR: Estimated glomerular filtration rate
FBG: Fasting blood glucose
FBI: Fasting blood insulin
GED: General educational development
GGT: Glutamyl transpeptidase
HbA1c: Glycosylated hemoglobin A1c
HDL-C: High-density lipoprotein cholesterol
HR: Hazard ratio
IR: Insulin resistance
LDL-C: Low-density lipoprotein cholesterol
LDH: Lactic dehydrogenase
CMS: Cardiometabolic syndrome
NCHS: National Center for Health Statistics
NCEP: National Cholesterol Education Program
NDI: National Death Index
NHANES: National Health and Nutrition Examination Survey
PIR: Poverty-income ratio
SBP: Systolic blood pressure
SCR: Serum creatinine
SD: Standard deviation
SUA: Serum uric acid
TBIL: Total bilirubin
TC: Total cholesterol
TG: Triglyceride
TyG: Triglyceride-glucose index
US: United States
